# Orthodontic Space Management and Mandibular First Premolar Autotransplantation Following Traumatic Anterior Tooth Loss in a Child with Five-Year Clinical and Radiographic Follow-Up: A Case Report

**DOI:** 10.3390/dj14070435

**Published:** 2026-07-13

**Authors:** Viviana-Elena Tesinschi, Pawel Plakwicz, Arina Vinereanu, Liviu Mirea, Loredana Golovcencu

**Affiliations:** 1 Orthodontics and Periodontology Service, Department of Orthodontics and Dentofacial Orthopaedics, Institut de Recherche Expérimentale et Clinique (IREC), University Clinic Saint-Luc, Catholic University of Louvain (UCL), 1200 Woluwé Saint-Lambert, Belgium; 2 Private Dental Practice “Dentalplan”, 00-443 Warsaw, Poland; info@plakwicz.com; 3 Department of Pedodontics, Faculty of Dentistry, Carol Davila University of Medicine and Pharmacy, 010232 Bucharest, Romania; arina.vinereanu@umfcd.ro; 4 Private Dental Practice, CMI Dr. Mirea Liviu, 010221 Bucharest, Romania; drliviumirea@yahoo.com; 5 Department of Orthodontics and Dentofacial Orthopaedics, Faculty of Dentistry, University of Medicine and Pharmacy, 700115 Iasi, Romania; loredana.golovcencu@umfiasi.ro

**Keywords:** orthodontic space management, traumatic tooth loss, autotransplantation, case report

## Abstract

Background/Objectives: The management of severe anterior dental trauma in growing patients remains a clinical challenge. This case report describes the treatment of a 10-year-old boy presenting with traumatic loss of maxillary anterior teeth following a road traffic accident, including avulsion of two permanent incisors and a primary canine, associated with luxation and intrusion of the contralateral central incisor. Methods: A multidisciplinary approach involving paediatric dentistry, orthodontics, oral surgery, and restorative dentistry was implemented. Treatment aimed to preserve the traumatised incisor and restore the anterior maxillary region through a biological approach combining mandibular first premolar autotransplantation and orthodontic space management. Results: The transplanted first mandibular premolar demonstrated favourable healing, with continued root development and a satisfactory crown-to-root ratio. After orthodontic treatment, the tooth was restored with a composite veneer, achieving good aesthetic and functional outcomes, maintained at the 5-year follow-up. Conclusions: This case highlights that autotransplantation combined with orthodontic space management may represent a viable treatment option in carefully selected growing patients, allowing preservation of alveolar bone, function, and aesthetics. Appropriate surgical, orthodontic, and restorative planning was crucial to the treatment’s success.

## 1. Introduction

Traumatic dental injuries (TDIs) represent a significant public health concern due to their high prevalence and impact on affected individuals. A landmark meta-analysis by Petti et al. [1] reports a global prevalence of TDI in permanent teeth of approximately 15.2%, with variations depending on geographic region [2,3] and age group [4]. Authors [1,3,4] found permanent maxillary incisors to be most frequently affected, and critical injuries such as avulsion and luxation are often associated with complex management and uncertain prognosis.

Anterior tooth loss during childhood represents a particular therapeutic challenge. Beyond the immediate aesthetic and functional impairment, prolonged absence of anterior teeth may compromise alveolar bone development and complicate future rehabilitation during craniofacial growth. While implant-supported rehabilitation is generally postponed until growth completion, removable prostheses often present functional, aesthetic, and compliance-related limitations.

Orthodontic space closure combined with biological tooth substitution offers an alternative approach that can preserve alveolar bone, maintain arch integrity, and avoid prolonged edentulous spaces. When suitable donor teeth are available, autotransplantation provides additional advantages through preservation of a functional periodontal ligament, allowing physiological bone remodelling and adaptation during growth.

This report describes the multidisciplinary management of severe anterior dental trauma in a growing patient through orthodontic space management and mandibular premolar autotransplantation, with a five-year follow-up demonstrating favourable functional, aesthetic, and dentoalveolar outcomes.

## 2. Case Presentation

**Figure 1:**
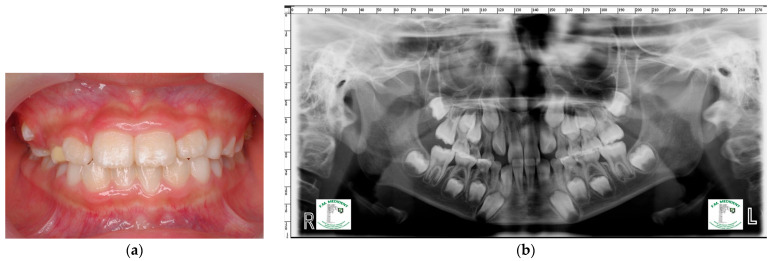
(**a**) Patient’s frontal occlusion one week prior to trauma; (**b**) panoramic X-ray one year prior to trauma, March 2018.

The patient, a 10-year- and 7-month-old Asian male at the time of a car accident, had been undergoing orthodontic treatment with removable appliances initiated prior to the trauma for maxillary constriction, anterior edge-to-edge occlusion, mandibular crowding, and a tendency toward skeletal Class III development. At the time of the accident, the incisors were aligned, the occlusion had been corrected, and the patient was in the mixed dentition phase with ongoing dental transition. Figure 1 shows the patient’s frontal occlusion and panoramic prior to the accident.

## 3. Clinical Findings

**Figure 2:**
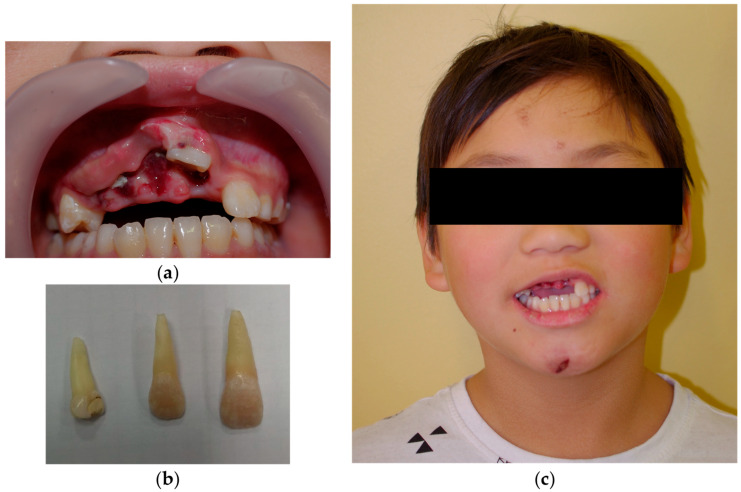
(**a**) Intraoral photo on the 4th day after the trauma; (**b**) the avulsed teeth; (**c**) facial appearance on the 4th day after the trauma. March 2019.

Following the accident, the patient sustained traumatic avulsion of teeth 53, 13, and 11, as well as vestibular luxation with intrusion of tooth 21, associated with fractures of the buccal alveolar bone. The patient was hospitalised for four days and received emergency management for craniofacial trauma, including tetanus prophylaxis and antibiotic coverage. No cerebral injury was identified, with the lesions being limited to the dento-alveolar and maxillary regions. The avulsed teeth were recovered late after the accident, and replantation was not attempted (Figure 2a,b).

The patient arrived at the orthodontist four days after the injury.

Extraoral exam showed a healing contused laceration in the mental region, frontal abrasion, no oedema, competent lips at rest, balanced face proportion but a slight asymmetry due to the loss of upper lip support. There was a significantly compromised facial aesthetic due to anterior maxillary tooth loss (Figure 2c).

Functional disturbances were observed, including impaired swallowing, altered phonation, reduced masticatory efficiency, and inability to perform incisal function due to anterior maxillary tooth loss.

At the intraoral exam, the anterior maxillary edentulous ridge appeared irregular, with recent post-traumatic extraction sockets containing blood clots. Tooth 21 presented buccal luxation associated with partial intrusion (Figure 2a).

**Figure 3:**
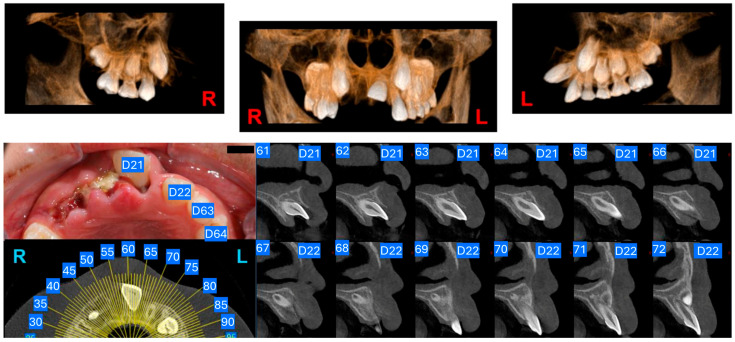
Post-traumatic CT scan showing vestibular luxation with intrusion of tooth 21 and fracture of the buccal alveolar bone, April 2019.

Radiologic examination: a CT was prescribed to analyse the state of tooth 21 and the alveolar bone. The CT confirmed the buccal luxation and intrusion and fracture of the buccal alveolar bone but happily no lesion on the root of the tooth (Figure 3).

Diagnosis: Post-traumatic anterior maxillary dentoalveolar injury with partial anterior maxillary edentulism involving teeth 11, 12, and 53; buccal luxation of tooth 21; enamel dystrophy of permanent teeth; Angle class I molar relationship; functional disturbances (swallowing, phonation, mastication, incisal function and aesthetic impairment with loss of upper lip support) secondary to anterior maxillary tooth loss.

## 4. Treatment Objectives

The accident resulted in maxillary anterior tooth loss due to the avulsion of the right maxillary incisors, combined with a guarded prognosis for tooth 21.

As this was a growing patient, the objectives were:

(1) Preservation of the luxated maxillary incisor (tooth 21) whenever possible.
(2) Maintenance of alveolar bone volume in the anterior maxillary edentulous area.
(3) Partial closure of the anterior edentulous space.
(4) Restoration of anterior aesthetics and function, including incisal function.
(5) Establishment of a stable dentoalveolar environment for long-term rehabilitation.

The treatment consisted of an initial emergency phase addressing the traumatised teeth, followed by a multidisciplinary oral rehabilitation aimed at minimising the long-term consequences of the injury on patient growth.

**Figure 4:**
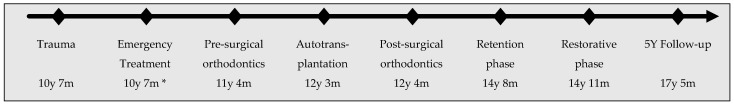
Timeline highlighting main treatment phases and corresponding patient age. * Emergency treatment performed 3 weeks post-trauma.

Management was multidisciplinary, involving a paediatric dentist, orthodontist, oral surgeon, and restorative dentist, and was carried out in several stages. The treatment timeline is shown in Figure 4.

1. Emergency management of the traumatised teeth was performed by the paediatric dentist.
2. Oral rehabilitation following the trauma, which included:
  (A) Orthodontic treatment in two phases: the first phase aimed to reposition the left maxillary central incisor affected by luxation, and the second phase focused on restoring the edentulous maxillary arch using biological substitution through autotransplantation of tooth 44 into the position of tooth 11, combined with medialization of the remaining teeth to close the edentulous space.
  (B) Surgical treatment, performed by an oral surgeon, consisting of autotransplantation using the right mandibular first premolar (tooth 44) as a donor tooth to replace the avulsed right maxillary central incisor.
  (C) Restoration of the transplanted tooth with a veneer replicating the morphology of the avulsed incisor, carried out by a restorative dentist after completion of orthodontic treatment.

Following completion of treatment, annual follow-up is required to monitor the transplanted tooth, the previously luxated incisor, as well as alveolar and skeletal growth.

## 5. Treatment Progress

### 5.1. Emergency Management of Traumatised Teeth

**Figure 5:**
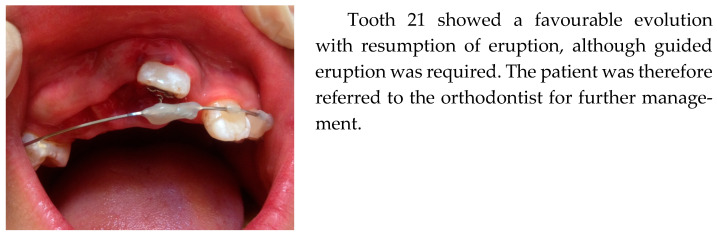
Tooth 21 after repositioning under anaesthesia and immobilisation, April 2019.

The emergency management was performed by a paediatric dentist and consisted of the following:

- Surgical repositioning of tooth 21 followed by monitoring of its spontaneous re-eruption. In the absence of spontaneous eruption, orthodontic traction was planned. Under local anaesthesia, a minimally invasive surgical repositioning of tooth 21 was performed. The tooth was stabilised using a semi-rigid splint consisting of a 0.018 × 0.022-inch stainless steel vestibular wire and a palatal bonded button with composite material. Teeth 14, 22, and 63 were also included in the semi-rigid splint using the same wire (Figure 5).
- Monitoring of root development and pulp vitality. As the intervention was performed three weeks after the traumatic event, the risks of ankylosis and external root resorption were increased. Therefore, the long-term prognosis of tooth 21 was considered guarded. However, maintaining the tooth in the arch was essential for preservation of the alveolar bone in view of future implant-supported rehabilitation after completion of skeletal growth. The patient had weekly follow-ups to monitor progress.

### 5.2. Orthodontic Phase of Oral Rehabilitation

At the time of trauma, the patient was 10 years and 7 months old and in the mixed dentition stage, with delayed dental development based on the discrepancy between the patient’s chronological age and the observed eruption stage. Mandibular primary canines and molars were still present, as well as the maxillary second primary molars and the first premolar on the traumatised side.

Two growth-related considerations were essential for treatment planning. First, the loss of maxillary incisors could negatively impact maxillary development, potentially aggravating a tendency toward mandibular prognathism due to insufficient maxillary growth, as suggested by the initial diagnosis. Second, implant-supported rehabilitation was not feasible at this stage and had to be postponed until completion of skeletal growth.

Three treatment approaches were considered:

(1) Prosthetic rehabilitation using a removable partial denture, which would be periodically modified following the eruption of the maxillary canine on the affected side and adjusted until completion of skeletal growth, to allow for future implant placement.
(2) Orthodontic space reduction to a single missing tooth using tooth 13 as a replacement for tooth 12, followed by fixed prosthetic rehabilitation using tooth 13 as an abutment with a mesial cantilever replacing tooth 11.
(3) Complete space closure through a biological approach using autotransplantation of tooth 44 in the position of tooth 11 combined with medialization of the remaining teeth to close the edentulous space.

During and after the orthodontic treatment, careful monitoring of both the previously luxated and intruded incisor and the autotransplanted tooth was required.

Removable prosthetic rehabilitation would have addressed aesthetic concerns and maintained space for future implant placement. However, in the absence of functional stimulation, significant alveolar bone resorption was expected during the prolonged period required before implant placement.

Considering that the patient still had several years of skeletal growth remaining, the option of space closure through biological substitution was considered more appropriate. In this context, preserving natural teeth to maintain functional stimulation of the alveolar bone was deemed more important, even in the presence of size discrepancies, particularly regarding the maxillary right canine, which was intended to substitute for the avulsed lateral incisor on that side.

The delayed dental age, together with the incomplete root development of the premolars (approximately two-thirds formed, Moorrees’ root development stage 3/4), supported the feasibility of autotransplantation. Therefore, an opinion was sought from an oral surgeon with experience in autotransplantation, based in another European Union country, who confirmed that this approach had a high likelihood of success.

The third option was selected in agreement with the patient’s parents.

### 5.3. Orthodontic Treatment—Pre-Surgical Phase

The objectives of the pre-surgical orthodontic treatment were:

- A conservative approach for tooth 21, aiming for its gradual repositioning while preserving pulp vitality.
- Reduction in the edentulous space through medialization of the canine into the position of the lateral incisor, followed by medialization of the posterior teeth on the affected hemiarch.

Three months after the trauma and before the initiation of orthodontic treatment, the patient was abroad for 3 months and wore a palatal plate with a space maintainer (Hawley with two prosthetic teeth in place of teeth 11 and 12).

**Figure 6:**
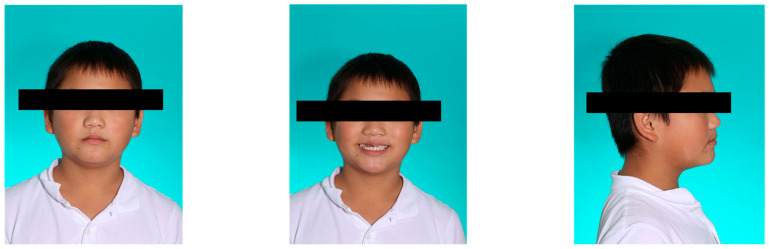
Facial photographs before the initiation of pre-surgical orthodontic treatment, November 2019.

**Figure 7:**
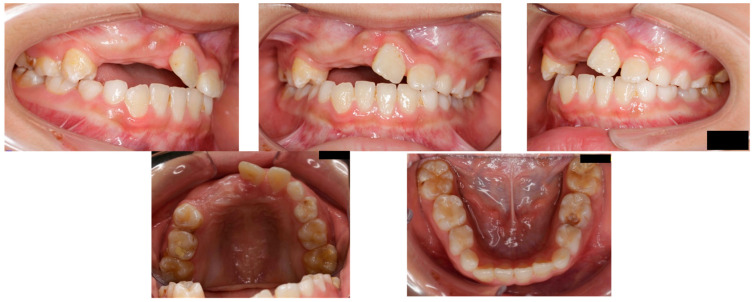
Intraoral photographs before the initiation of pre-surgical orthodontic treatment, November 2019.

**Figure 8:**
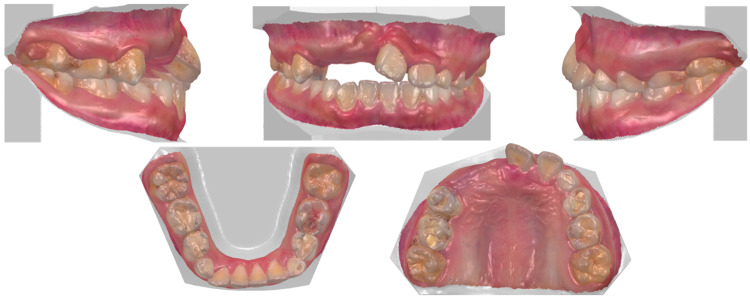
Digital casts before the initiation of pre-surgical orthodontic treatment, November 2019.

After the patient’s return, radiographs, study models, and clinical photographs were obtained (Figure 6, Figure 7 and Figure 8). The orthodontic records were then sent to the oral surgeon to determine the most suitable donor tooth for autotransplantation.

Extraoral examination showed well-balanced facial proportions. However, flattening of the upper lip contour on the right side persisted because of tooth loss. The facial profile was straight (Figure 6).

Intraoral examination revealed a continued delay in dental eruption. In the mandible, the dental formula remained unchanged compared to the post-trauma situation. A slight flattening of the incisal curvature was observed. In the maxilla, on the affected right hemiarch, the first premolar was present, with no significant changes in dental eruption compared to the post-trauma status. The edentulous alveolar ridge had healed well and presented adequate width. Tooth 21 remained in protrusion and was rotated mesiobuccally with a root distal tipping. The molar relationship was Angle Class I, and the maxillary and mandibular midlines were coincident (Figure 7 and Figure 8).

**Figure 9:**
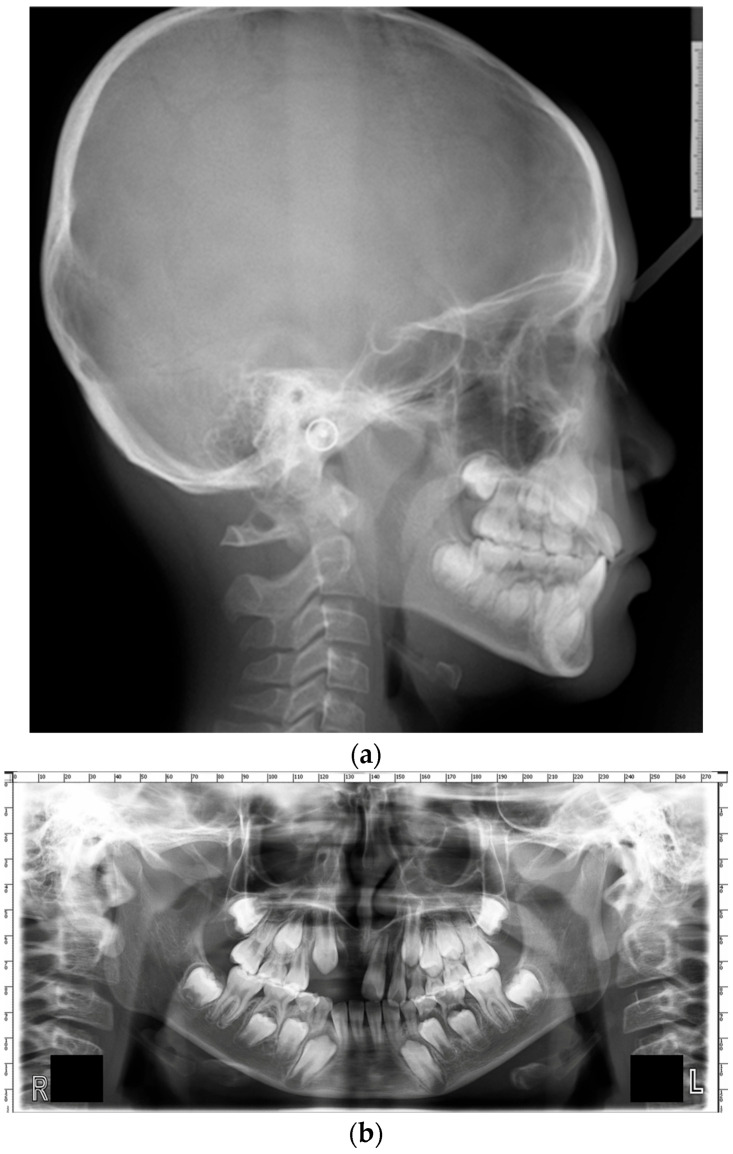
Cephalometric (**a**) and panoramic (**b**) X-rays before the initiation of pre-surgical orthodontic treatment, November 2019.

Panoramic radiography showed that tooth 13 had initiated intraosseous mesial movement. The premolar roots were approximately half developed, and root resorption of the second primary molars was more advanced in quadrant 3. Third molar germs were not yet visible (Figure 9b).

Cephalometric analysis showed a skeletal Class I relationship, although the ANB angle was slightly below 2°, suggesting a borderline tendency toward Class III. A hypodivergent growth pattern was observed. The lower incisor inclination was 89°, which is relatively uncommon in Asian populations, indicating a dental compensation tendency toward a Class III pattern that needed to be considered during treatment planning (Figure 9a, Table 1).

Preparation of the recipient site for autotransplantation required 1 year and 4 months and consisted of creating a 10 mm space between teeth 13 and 21. The donor tooth (44) had a mesiodistal width of approximately 8 mm, requiring an additional 1 mm of space medially and distally relative to the adjacent teeth.

This space was achieved using a fixed orthodontic appliance (Discovery Roth 0.018 system, Dentaurum) in the maxilla. At the end of this phase, tooth 13 had substituted for tooth 12, tooth 14 had substituted for tooth 15, and group medialization was achieved in the first quadrant. Before surgery, a passive 0.016 × 0.025-inch stainless steel stabilising archwire was inserted. Closed-coil springs were used to maintain the spaces created for transplantation, while braided ligature ties were placed to preserve the maxillary midline.

### 5.4. Surgical Phase

**Figure 10:**
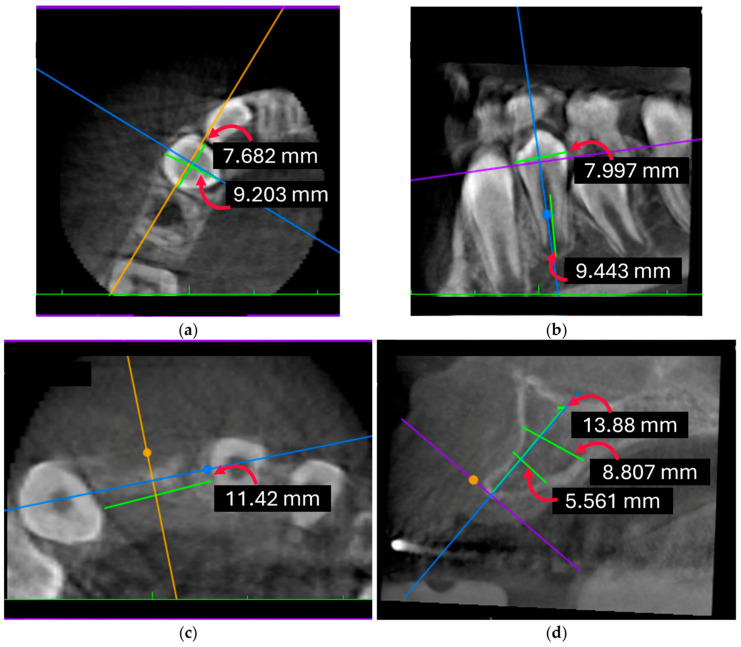
CT scans performed before surgery (December 2020): donor 44 axial section (**a**), donor 44 sagittal section (**b**), recipient site 11 axial section (**c**), and recipient site 11 sagittal section (**d**). Relevant dimensions of donor and recipient site are shown as green lines, with corresponding measurements.

Prior to the surgical intervention, the oral surgeon performed a new CT scan (Figure 10), based on which it was decided to use tooth 44 as the donor tooth for replacement of tooth 11, as its morphology was considered more favourable than that of tooth 45 (smaller lingual cusp).

**Figure 11:**
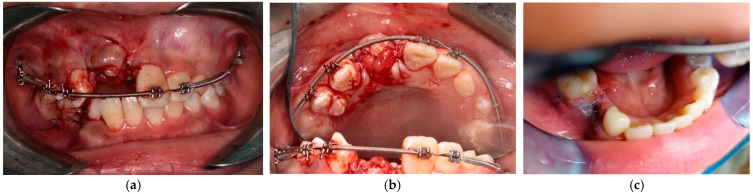
(**a**,**b**) Autotransplanted tooth immediately after surgery (December 2020); (**c**) mandibular donor site.

The surgery was performed under local anaesthesia. The flap in the anterior maxilla was created by incision of the interdental papillae at teeth 13 and 21 and elevated with no vertical incisions. The surgical socket was prepared with a set of three surgical burs (of 4, 6 and 7 mm diameter). There was distinct labial dehiscence of alveolar bone due to a narrow alveolar ridge at the site of the lost right incisors. In the mandible the anaesthesia was also limited to local infiltration at the site of donor tooth 44 and primary tooth 84. The primary molar was extracted with forceps. The surgical access was performed by creating a flap through the interdental papillae and a vertical vestibular incision at tooth 85. The buccal bone was gently removed with burs under copious irrigation with saline to expose the coronal part of donor tooth 44. Then, the donor was gently elevated from its bone crypt and transferred to the prepared surgical socket at site 11. The soft tissue of the dental follicle was left intact on the crown of the donor. The donor was stabilised using only braided and coated polyglycolic acid synthetic absorbable 5-0 sutures coming through the gingiva and crossing over the central sulcus of the donor’s crown. No additional rigid splinting was applied. The donor site was also closed with absorbable 5-0 sutures. The patient was administered ibuprofen and paracetamol every 8 h for two days and prophylaxis with amoxicillin 457 mg taken twice daily for seven days. Chlorhexidine gel was administered topically three times daily for two weeks. Figure 11 depicts the transplant and donor sites immediately after surgery.

A postoperative control was carried out one day after surgery, showing a favourable clinical evolution. The patient then returned to their country of residence and continued follow-up at the orthodontic clinic, under combined monitoring by the orthodontist locally and the oral surgeon remotely.

### 5.5. Orthodontic Treatment—Post-Surgical Phase

Two weeks after surgery, the sutures were removed, and the transplanted tooth was allowed to erupt spontaneously. Standard post-autotransplantation follow-up included periapical radiographs at 1, 3, 6, and 12 months, followed by annual controls, as well as pulp vitality testing.

**Figure 12:**
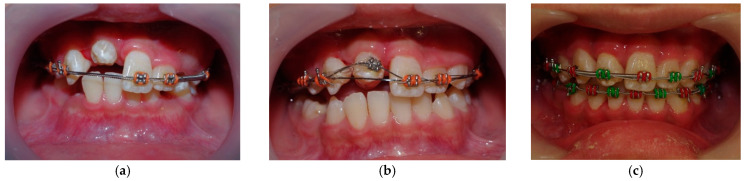
(**a**) Transplanted tooth at 1-month post-surgery (January 2021); (**b**) transplanted tooth at 3 months post-surgery (March 2021); (**c**) transplanted tooth at the end of orthodontic treatment (December 2022).

The one-month postoperative periapical radiograph showed favourable root development, with no signs of resorption. Clinically, the tooth had resumed eruption and presented physiological mobility (Figure 12a).

At three months post-surgery, eruption had ceased, mobility had decreased, and the percussion sound had changed, these clinical findings suggesting a possible transient ankylosis. Given the early postoperative stage, no additional CBCT examination was performed. Gentle orthodontic traction was therefore initiated to encourage eruption and assess the biological response of the transplant. A bracket for tooth 12 was used for the autotransplant 44 and it was incorporated into the fixed orthodontic appliance. Light traction was applied by orthodontic traction using a 0.010-inch NiTi overlay archwire, followed by a 0.012-inch NiTi overlay, both placed over a 0.016 × 0.022-inch stainless steel base arch. Because of the reduced mobility of the transplanted tooth, gentle functional loading (biting on a wooden stick) was recommended until eruption resumed. This recommendation was empirical and reflected the treating surgeon’s clinical practice, but there is experimental evidence suggesting that functional stimulation may promote periodontal ligament healing and reduce ankylotic healing after transplantation [5,6]. After five months of exercises combined with light orthodontic traction, eruption resumed, and physiological mobility was restored. (Figure 12b).

Once spontaneous eruption of tooth 44 had resumed, treatment proceeded with a conventional archwire sequence consisting of 0.014-inch NiTi, 0.016-inch NiTi, 0.016 × 0.022-inch NiTi, 0.016 × 0.022-inch stainless steel, and 0.016 × 0.025-inch stainless steel archwires (GAC). The placement of the mandibular fixed appliance, one year after the maxillary appliance, was postponed until completion of dental eruption and exfoliation of the primary teeth.

Space closure in the first quadrant was achieved through posterior-to-anterior mesialization using compressive coil springs and Class III elastics. In the fourth quadrant, mesial movement of tooth 46 was primarily obtained by taking advantage of the eruptive potential of tooth 47. To maintain the maxillary and mandibular dental midlines, passive ligature ties were placed beneath the archwire between teeth 21–26 and 31–36, respectively. Appropriate torque expression was obtained by using a maxillary lateral incisor bracket (tooth 12 prescription) for the transplanted tooth 44 and a canine bracket (tooth 13 prescription) for tooth 14. Although orthodontic extrusion of tooth 13 could have been performed to create a narrower cervical contour more characteristic of a maxillary lateral incisor, this would have necessitated more extensive reshaping of the canine crown. Following discussion of the available options, the patient and parents were satisfied with the existing canine appearance and declined further aesthetic modification. Therefore, only limited enameloplasty of the cusp tip and the mesial and distal surfaces was performed. The total duration of orthodontic treatment was 3 years and 3 months (Figure 12c).

The radiologic follow-up consisted of periapical radiographs at 6, 9, 12, 18, and 24 months and annual panoramic radiographs.

### 5.6. Orthodontic Treatment Results

**Figure 13:**
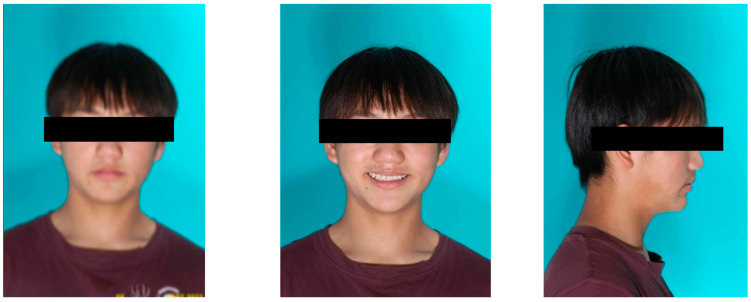
Facial photographs at the end of the post-surgical orthodontic phase, July 2023.

At the end of orthodontic treatment, the facial thirds were well balanced, and lip symmetry was restored due to the dental support achieved through closure of the edentulous space. The profile became slightly convex and facial aesthetics were restored (Figure 13).

**Figure 14:**
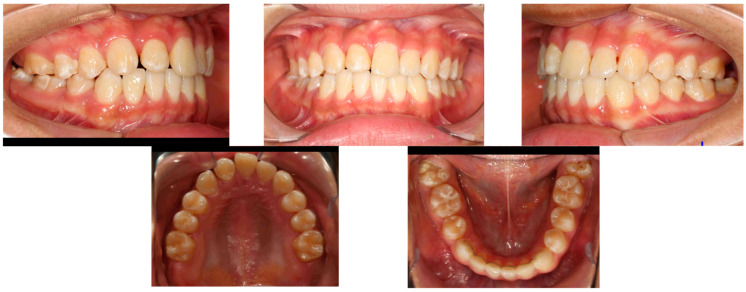
Intraoral photographs at the end of the orthodontic treatment, July 2023.

**Figure 15:**
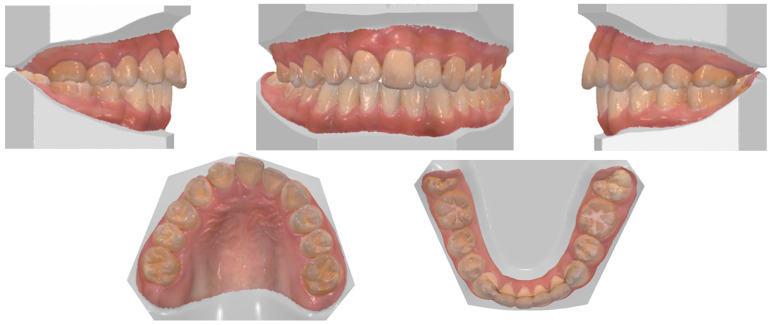
Dental casts at the end of the orthodontic treatment, July 2023.

Intraorally, the spaces resulting from the loss of the maxillary incisors and the extraction of the premolar in the fourth quadrant were closed, except for two small spaces intentionally maintained for the restoration of the transplanted tooth. The second permanent molars were still erupting at that stage. There was a class I Angle molar relationship, a good inter arch coordination and a stable occlusion. The maxillary and mandibular midlines were coincident. However, both dental arches showed slight transverse and sagittal asymmetries, related to the absence of one dental unit on each of the right maxillary and mandibular hemiarches (Figure 14 and Figure 15).

**Figure 16:**
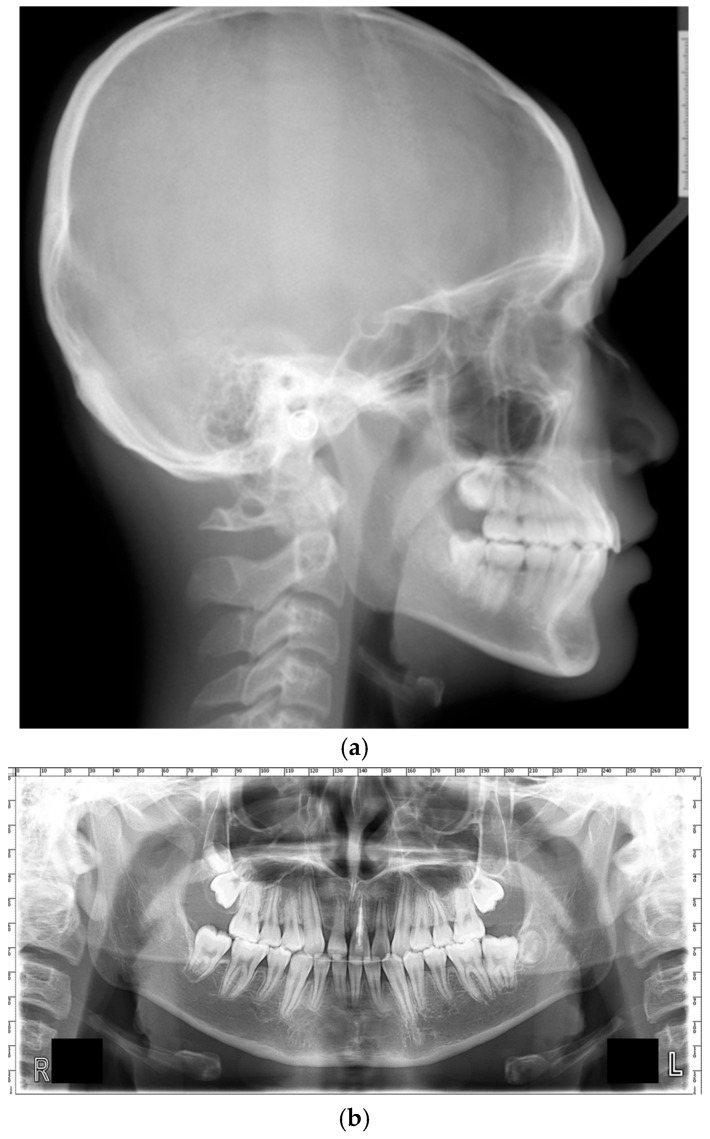
Cephalometric (**a**) and panoramic (**b**) X-rays at the end of the orthodontic treatment, July 2023.

The panoramic radiograph confirmed successful space closure and satisfactory root parallelism. All permanent second molars were present, although the maxillary second molars were still erupting and remained in a relatively high position. Developing mandibular third molar germs were visible in quarters 1 and 3 (Figure 16a). The cephalometric radiograph demonstrated favourable maxillary growth, resulting in a slight increase in the ANB angle (Figure 16b). The inclination of the incisors had normalised (Table 2).

Retention consisted of a fixed retainer in the mandible and Essix retainers for both the maxilla and mandible. The maxillary retainer was worn until restoration of the transplanted tooth with a composite veneer. Subsequently, a fixed maxillary retainer was placed, and a new maxillary Essix retainer was fabricated.

The transplanted tooth showed a very favourable outcome: root development was completed, with a crown–root ratio greater than 1 and a root length comparable to that of the other premolars. Moreover, pulp sensitivity thermal tests were positive, although with progressively decreasing intensity over time, consistent with pulp canal obliteration, a phenomenon well described in the literature [7,8,9,10,11].

**Figure 17:**
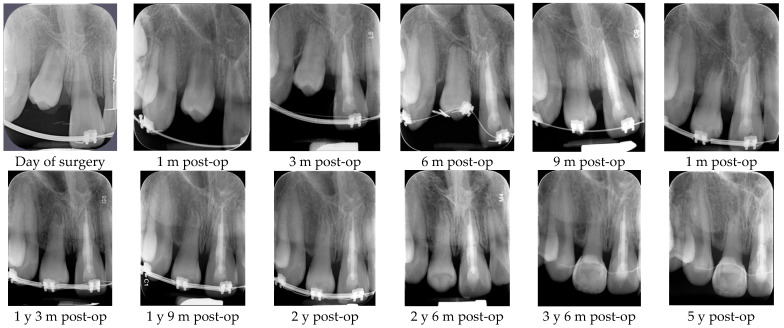
Sequential periapical radiographs of tooth 44 in the five years following the autotransplantation. Time elapsed in years (y) and months (m). Dimensions: 3.05 × 4 cm.

**Figure 18:**
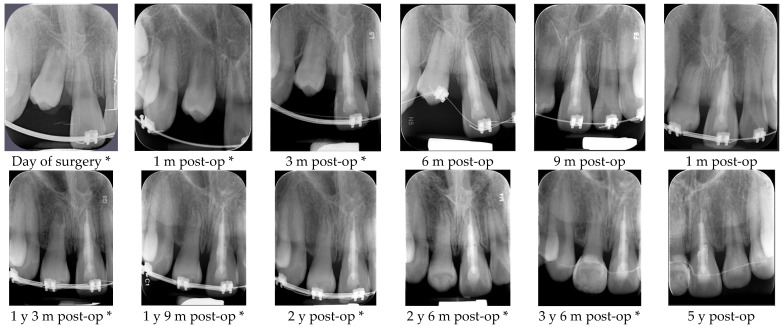
Sequential periapical radiographs of tooth 21 in the five years following the autotransplantation. Due to a lack of clearer radiographs, some are copies of the entries in Figure 17, marked with *. Time elapsed in years (y) and months (m). Dimensions: 3.05 × 4 cm.

Regarding tooth 21, which had sustained buccal luxation and total intrusion and was surgically repositioned, there are some common consequences of this type of trauma: inflammatory root resorption, ankylosis, pulpal necrosis, and loss of marginal bone support [12,13]. A localised external root resorptive defect was identified radiographically about 10 months after the traumatic event. In the absence of clinical signs of pulpal pathology, a conservative, vitality-preserving monitoring approach was adopted. CBCT examination performed prior to the autotransplantation procedure also revealed a periapical lesion associated with tooth 21. Following two consecutive weekly checkups showing negative thermal pulp sensibility testing, endodontic treatment was undertaken and pulpal necrosis was confirmed during canal instrumentation. The root canal was subsequently obturated with gutta-percha and a bioceramic sealer (BioRoot RCS, Septodont). The lesion and the transplanted tooth were monitored radiographically (Figure 17 and Figure 18). However, the long-term prognosis of this tooth remains guarded, with a risk of either ankylosis or progression of external root resorption.

Both the transplanted tooth and tooth 21 were to be monitored annually. Even if ankylosis or resorption were to occur, replacement with an implant would be postponed until an appropriate stage, after the completion of growth.

The crown-to-root ratio of the transplanted tooth was assessed on serial periapical radiographs up to the 2.5-year follow-up. Measurements were performed using the Inkscape software version 1.2.1 by determining the radiographic crown length and root length on standardised periapical images. Subsequent radiographs were not included in this analysis because a composite veneer was placed after the 2.5-year follow-up, modifying the clinical crown dimensions and preventing reliable comparison over time. Crown length was defined as the distance from the incisal edge to the cemento-enamel junction, and root length as the distance from the cemento-enamel junction to the root apex. Measurements were performed digitally on periapical radiographs using the software Inkscape. Minor variations between time points may reflect differences in radiographic projection and magnification. To minimise the influence of radiographic magnification, the crown-to-root ratio rather than absolute linear measurements was used. Owing to the retrospective nature of the case and the use of conventional radiographs, measurements should be interpreted as descriptive rather than absolute values. The crown-to-root ratio improvement from 1:1.03 at baseline to 1:1.93 after 2.5 years reflects substantial post-transplant root development (Table 3).

### 5.7. Restoration on the Buccal Surface of the Graft

Orthodontic treatment was followed by the last phase of oral rehabilitation, the restorative management performed by a specialist in endodontics and restorative dentistry, consisting of reconstruction of the buccal surface of the transplanted tooth to resemble the avulsed central incisor. Preparation for restoration involved maintaining small spaces medially and distally relative to the adjacent teeth, as well as positioning the graft in infraocclusion. After removal of the fixed appliances, the patient received an Essix retainer in the maxilla and fixed retention and an Essix retainer on the mandible and was referred to the restorative dentist. Following informed consent from the patient and parents, a direct composite veneer was planned to improve the morphology and aesthetics of the transplanted premolar replacing the maxillary central incisor, maintaining the specific aspect of the dystrophic enamel of the avulsed incisor. A preliminary impression was obtained and a diagnostic cast was fabricated. A wax-up was performed to define the final tooth morphology. Based on the wax-up, a silicone putty index (Speedex^®^, Coltene—Altstatten, Switzeland) was fabricated and used as a guide for reconstruction of the palatal surface and incisal edge.

Minimal preparation of the vestibular surface was performed, limited to approximately 0.2 mm. The palatal aspect of the restoration was first built using the silicone index as a guide. After placement of a rubber dam (Hygienic^®^, Coltene—Altstatten, Switzeland), the vestibular aspect was completed using a stratified direct composite technique.

The enamel surface was etched with 35% phosphoric acid (Ultra-Etch^®^, Ultradent—Salt Lake city, UT, USA) for 30 s, rinsed, and gently air-dried. A universal adhesive system (All-Bond Universal^®^, BISCO) was applied according to the manufacturer’s instructions and light-cured. The restoration was then incrementally built using enamel and flowable composite resins (Aelite Aesthetic Enamel^®^, Aelite Flow^®^, Bisco—Chicago, IL, USA), with each layer light-cured for 20 s.

**Figure 19:**
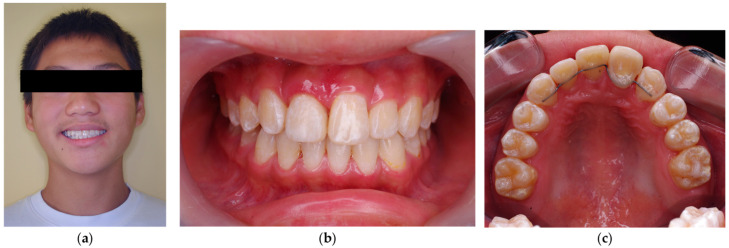
Facial photo (**a**), facial occlusion photo (**b**) and fixed retention on the maxilla (**c**) after composite restoration of the autotransplant, August 2023.

Finishing and polishing were performed using abrasive discs, polishing rubbers, and diamond polishing paste Diapolisher paste GC. Occlusion was carefully checked and adjusted to ensure functional integration of the restoration (Figure 19a–c).

### 5.8. Post-Retention Follow-Up

After completion of graft restoration and the retention phase, the patient was reviewed at annual follow-up visits. Periapical radiographs (Figure 17 and Figure 18) were obtained yearly to assess the condition of the roots of both the transplanted tooth and the incisor that had been orthodontically repositioned following trauma.

**Figure 20:**
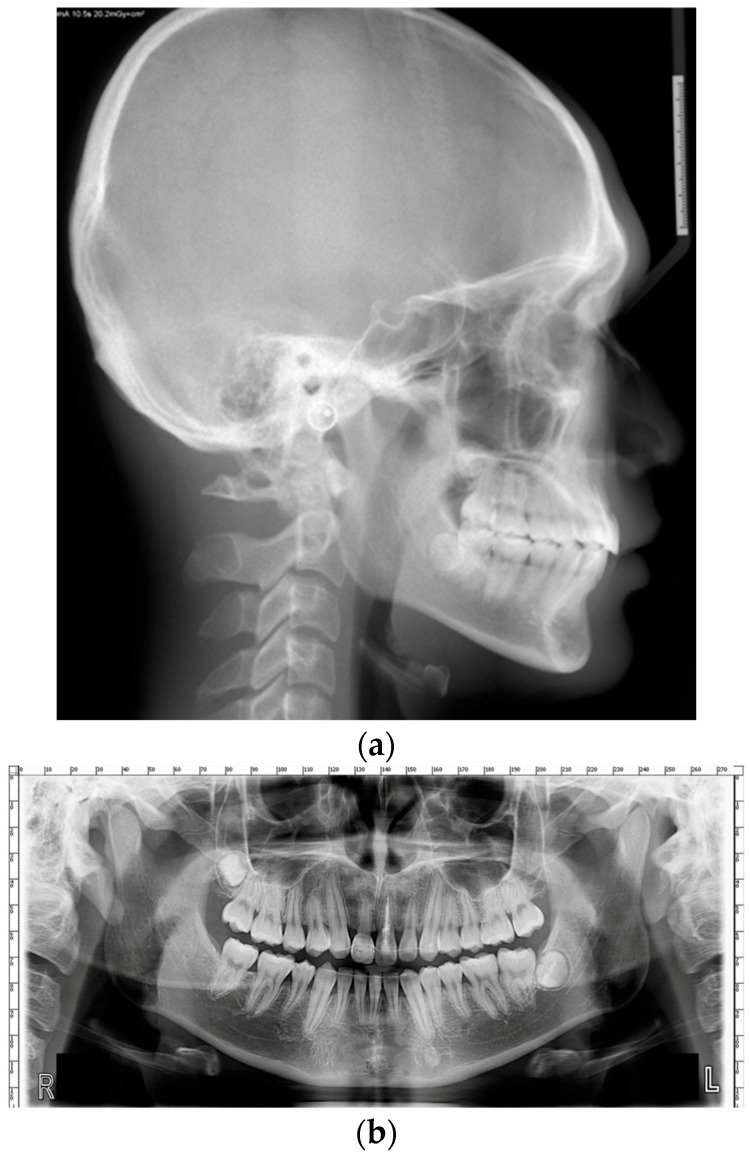
Cephalometric (**a**) and panoramic (**b**) X-rays five years after autotransplantation, December 2025.

At the latest follow-up, 5 years after autotransplantation, the patient was 17 years old. The second permanent molars had fully erupted, while the third molar germs (18 and 38) were present but lacked sufficient space in the dental arches. Panoramic, cephalometric (Figure 20a,b) and periapical (Figure 17 and Figure 18) radiographs and photos were taken.

**Figure 21:**
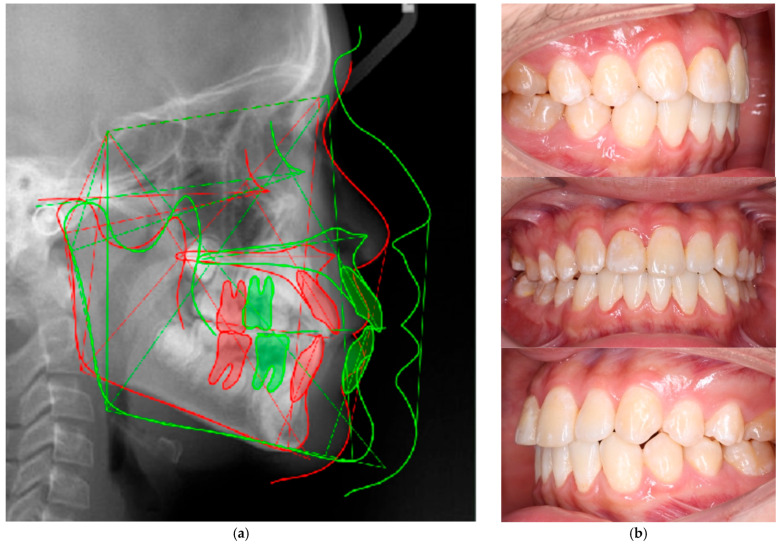
(**a**) Superimposition of cephalometric trays before treatment (red) and 5 years after the autotransplant (green) (**b**) photos of the occlusion 5 years after the autotransplant, December 2025.

The transplanted tooth showed complete root development, with a root length nearly equal to that of its mandibular counterpart. Partial pulp canal obliteration was observed, with no signs of resorption (Figure 17 and Figure 18). The tooth exhibited normal mobility, and the orthodontic treatment results remained stable (Figure 21b).

**Figure 22:**
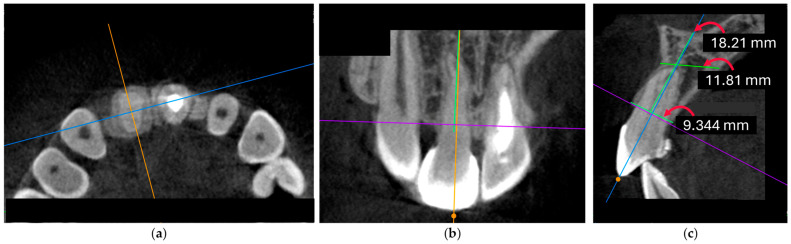
Axial (**a**), coronal (**b**), and sagittal (**c**) CT scans taken 7 years after trauma, at the 5-year post-transplantation follow-up, May 2026. The height of the alveolus increased by 5 mm. Subfigure (**c**) highlights in green the dimensions of the alveolar bone.

Regarding the previously luxated incisor, no significant clinical changes were observed on the periapical radiograph during the first 4 years after the trauma. However, the most recent periapical radiograph, taken 5 years after the trauma, revealed changes in the cervical lesion that require further evaluation (Figure 18, last). Therefore, the patient was referred for a comprehensive endodontic re-assessment and CBCT examination of tooth 21. A CBCT examination performed seven years after the trauma and three months after the last periapical radiograph demonstrated radiographic improvement of the previously observed resorptive lesion on the distal root surface of tooth 21, suggesting stabilisation of the lesion following endodontic treatment (Figure 22). The transplanted tooth exhibited a continuous periodontal ligament space around the root surface.

Two years after completion of treatment, the patient experienced a prepubertal growth spurt. Significant mandibular growth was observed, balanced by corresponding maxillary development, despite the initial skeletal Class III tendency and the orthodontic outcome has remained stable (Figure 21a). The increase in SNA, together with the maintenance of a favourable ANB relationship, suggests that the treatment approach successfully preserved the growth potential of the anterior maxillary region while avoiding the alveolar deficiencies commonly associated with prolonged anterior edentulism during childhood. An autotransplanted tooth maintains a functional periodontal ligament, allowing physiological bone remodelling and adaptation to craniofacial growth [14]. The presence of natural teeth within the anterior maxillary segment may also have contributed to the preservation of alveolar bone volume (measured on the last CT) and continued dentoalveolar development [15]. Between treatment completion and the 5-year post-autotransplantation follow-up, the mandible resumed its anterior rotational pattern. The interincisal angle decreased slightly due to mild labial inclination of the incisors in both arches (Table 4, Figure 20a).

## 6. Discussion

The management of post-traumatic loss of anterior teeth in growing patients can be achieved through several treatment approaches, including dental replantation, provisional prosthetic rehabilitation to maintain space for future implant-supported restoration after completion of skeletal growth, and orthodontic tooth substitution through space closure or autotransplantation. In the present case, all these treatment options were carefully analysed in terms of their advantages and limitations.

Replantation of an avulsed permanent tooth is indicated under specific conditions, including an intact tooth with a viable periodontal ligament, minimal extra-oral dry time (less than 15–20 min, or up to 60 min if stored in an appropriate medium such as saline, milk, or saliva), and the absence of medical contraindications, as recommended by the IADT guidelines and survey studies [16,17]. Although favourable survival rates have been reported, long-term complications such as ankylosis and external root resorption remain frequent. In the present case, the avulsed teeth were recovered after 3 days and were not stored in a suitable medium; therefore, this option was excluded from the start.

Removable prostheses can provide a temporary aesthetic and functional solution after traumatic avulsion of permanent incisors in children. The prosthetic replacement may involve acrylic or composite teeth, and in certain situations, the use of natural crowns from avulsed teeth. However, their use is limited by issues related to patient compliance, retention, hygiene, and potential interference with growth. In addition, the lack of functional stimulation may lead to progressive alveolar bone resorption during the prolonged period required before implant placement. If this kind of space maintenance had been chosen with the intention of later replacing the two missing incisors with an implant-supported restoration, it would have had to be worn until the completion of growth, all throughout adolescence. However, even when preceded by alveolar ridge augmentation, the placement of two adjacent implants in the anterior maxilla remains an aesthetic challenge. Optimal papillary fill between two implants cannot always be achieved. Therefore, reducing the edentulous space to a single tooth replacement is generally considered a more favourable option from an aesthetic perspective [18,19]. In the present case, the patient already exhibited a tendency toward a Class III growth pattern, which could have been further accentuated by the loss of the maxillary incisors. Furthermore, the guarded prognosis of tooth 21 made this option less favourable, as repeated insertion and removal of a prosthesis could have increased the risk of its premature loss.

Preservation of alveolar bone volume represents a major challenge in growing patients with anterior tooth loss. Functional stimulation provided by natural teeth plays a key role in maintaining bone volume, whereas prosthetic solutions do not offer the same biological advantage.

Fixed prosthetic rehabilitation was also considered. The literature describes the use of Maryland-type bridges [20], as well as orthodontic temporary anchorage devices (TADs) as interim abutments for crown restoration [21,22,23]. Resin-bonded fixed dental prostheses require minimal enamel preparation and have demonstrated favourable clinical longevity extending over several years, as shown in systematic reviews and meta-analyses [20]. Moreover, long-term studies on mainly adult populations performed by Kern et al. demonstrate 98.2% and 97.3% survival rates after 10 and 15 years, respectively [24,25]. In the present case, this option would have been feasible only after orthodontic reduction in the edentulous space to a single tooth.

Although cantilever resin-bonded Maryland bridges have shown excellent long-term survival, they remain a prosthetic solution. In young patients, they are often used as a long-term interim treatment until craniofacial growth is complete and implant placement for the lost incisor becomes feasible [26]. Some research indicates that the aesthetic result for single implants in the aesthetic zone is suboptimal even for implants placed after the skeletal growth is complete because they could develop progressive infraocclusion over time [27].

A cantilever design with tooth 13 as support was considered given the improved survival and fewer technical complications compared with one fixed on both tooth 13 and 21.

Orthodontic TADs may provide a minimally invasive and reversible approach to interim tooth replacement, particularly for single missing incisors. Different placement techniques have been described, including transmucosal occlusal gingival position and lingual positioning [21]. However, in the present case, the edentulous space resulted from the loss of three teeth, making this approach less suitable. In addition, implant-supported solutions in growing patients remain temporary and are generally limited to single-tooth replacement. However, cases where such implants have remained in place from 12 months up to 99 months have been reported, with favourable stability and minimal complications reported [23].

Orthodontic space closure through mesial movement of adjacent teeth has been described as a viable approach for managing anterior edentulous spaces. Case selection depends on factors such as malocclusion, dental and skeletal age, and the need for tooth reshaping or extractions [28].

Several case reports have described successful outcomes of maxillary lateral incisors that replaced central incisors [22,28]. In the present case, this approach involved substitution of the lateral incisor with the canine, followed by medialization of the remaining teeth, resulting in a single-tooth space at position 11 and a shortened dental arch. Long-term studies regarding the substitution of congenitally missing lateral incisors for canines and the substitution of canines for first premolars may lead to an acceptable functional relationship, good TMJ function and good periodontal outcome [29,30].

Autotransplantation of a premolar to replace an avulsed permanent maxillary incisor represents one of the main indications described by Slagsvold and Bjercke [31], pioneers of this treatment approach. A premolar at Moorrees’ root development stages 3 or 4 is considered the most suitable donor tooth, with reported success rates of approximately 85.4% and survival rates approaching 90% in various studies [7,8,10,31,32,33]. Success is defined by the presence of healthy periodontal hard and soft tissues, absence of progressive root resorption, and a crown-to-root ratio of less than 1.

In the present case, delayed dental development and the Moorrees’ stage between 3 and 4 with an open apex of the mandibular left premolars as donors allowed for autotransplantation. Autotransplantation was preferred over a Maryland cantilever bridge, as it retains a functional periodontal ligament and participates in alveolar development during growth [14,34,35]. Moreover, the biologic substitution by autotransplantation and space closure was the most cost-effective alternative.

A consultation with an experienced oral surgeon confirmed the feasibility of this approach. Although the option of replacing both incisors through transplantation was considered, a single autotransplantation at position 11 was selected due to logistical pandemic travel constraints and to minimise the risk of treatment failure. A donor tooth from quadrant 4 was selected to compensate for the absence of a tooth in quadrant 1 and to achieve a balanced occlusion at the end of treatment. Tooth 44 was preferred due to its more favourable morphology, with a smaller lingual cusp compared to tooth 45.

The duration of orthodontic treatment prior to autotransplantation was influenced by the initially high position of the permanent canine germ on that side. However, at the end of this stage, the premolar still presented approximately two-thirds of root development, which corresponds to the optimal stage for transplantation.

Following surgery, the transplanted tooth was reviewed at 1, 3, 6 and 12 months and annually thereafter. At each visit, clinical examination included assessment of tooth mobility, percussion sound, and pulp sensitivity thermal test, while periapical radiographs were performed to monitor pulpal healing and root development. Panoramic radiographs were taken annually, and a cephalometric radiograph was obtained at the 5-year follow-up.

Pulp healing was evaluated through the progressive development of pulp canal obliteration, which was observed approximately 6 months after transplantation, in accordance with findings reported in the literature [9,33,36]. Following the systematic review in Vinagre et al. [37], a “watchful waiting” approach was employed in the management of pulp canal obliteration, appropriate to cases without periapical pathology. Potential postoperative complications involve the pulp—namely pulp necrosis—and root development, including root resorption and ankylosis [7,9]. Root resorption usually occurs within the first two months after surgery, while ankylosis is generally diagnosed within six months following the procedure. In this case, during the early postoperative phase, the transplanted tooth initially showed favourable evolution, with spontaneous eruption and physiological mobility. Spontaneous eruption of the transplanted tooth is preferred, and orthodontic treatment should be postponed until pulpal and periodontal healing has occurred and before complete pulp canal obliteration, typically within 3 to 6 months after transplantation [7,9].

In this case, at 3 months, eruption ceased, mobility decreased, and the percussion sound changed—clinical signs suggesting transient ankylosis. The diagnosis could not be confirmed radiographically, as CBCT imaging was not considered justified at this early stage. Experimental studies have suggested that limited ankylotic areas may undergo spontaneous resolution through periodontal ligament regeneration [38]. Nevertheless, given the patient’s age and the importance of maintaining eruption and alveolar development, a decision was made to initiate gentle orthodontic traction rather than adopt a purely observational approach. Orthodontic traction had to be initiated, and the patient was instructed to perform daily mobilisation exercises. After approximately 5 months, eruption resumed, and physiological mobility was restored. Having already initiated the orthodontic traction, the exact contribution of the mobilisation exercises to the resumption of the eruption remains uncertain.

At long-term follow-up, the transplanted tooth demonstrated complete root development, with a favourable crown-to-root ratio and absence of root resorption. Partial pulp canal obliteration was present, as expected. The tooth remained functional, with normal mobility and stable integration in the dental arch.

Positioning the canine in the lateral incisor site may have aesthetic consequences due to differences in tooth shape and shade. In the present case, the size of the canine was close to that of the contralateral lateral incisor, and the presence of enamel dystrophy affecting all teeth reduced the visual impact of this substitution. In addition, the restorative dentist placed a composite veneer designed to closely reproduce the appearance of the altered enamel, resulting overall in a satisfactory aesthetic outcome and restoration of the patient’s smile.

Minimal stripping was performed on the canine, and the cusp tip was only slightly re-shaped to approximate the morphology of a lateral incisor.

Regarding restoration of the transplanted tooth with a central incisor veneer, there are good outcomes reported in the literature [10]. In this case the cervical dimension of the graft was smaller than that of a natural central incisor, and the clinical crown of the contralateral central incisor was larger than the veneer that could be fabricated for the graft. Despite these minor discrepancies, when considering the initial clinical situation and the outcome, the result can be regarded as favourable and likely to remain stable for many years.

The management of this case was further complicated by the COVID-19 pandemic, which imposed limitations on patient mobility and interdisciplinary coordination. Despite these constraints, a favourable outcome was achieved.

Annual follow-up is required for both the transplanted tooth and the external root resorption affecting tooth 21. Five years after the trauma, the resorption progressed very little and finally stabilised. In adulthood, the composite veneer on the transplanted tooth may be replaced with a ceramic veneer. Even if resorption or ankylosis were to occur in either of these teeth, extraction and replacement with an implant would be postponed as long as possible to allow completion of alveolar growth.

Autotransplantation has been shown to provide long-term survival, with studies reporting favourable outcomes over periods exceeding 10–15 years, while preserving alveolar bone volume for potential future implant placement [8,11]. This approach allows maintenance of functional and aesthetic stability during growth and adolescence.

The patient reported a positive experience throughout the treatment and adhered well to all clinical instructions. No concerns or objections were expressed during therapy. The patient’s family was highly supportive, which contributed to good compliance. Both the patient and the family expressed a high level of satisfaction with the treatment outcomes, particularly regarding comfort, oral function, and dental aesthetics.

This report describes a single clinical case, and long-term outcomes beyond adolescence remain to be evaluated. The success of this approach is dependent on patient compliance and interdisciplinary coordination, which may limit its reproducibility.

## 7. Conclusions

This case illustrates the successful conservative management of post-traumatic anterior tooth loss in a growing patient through a multidisciplinary approach. Effective interdisciplinary collaboration and adherence to treatment protocols contributed to a favourable functional and aesthetic outcome. Although conclusions cannot be generalised from a single case, this report suggests that autotransplantation combined with orthodontic space closure may be considered a treatment option in carefully selected growing patients when supported by appropriate surgical, orthodontic, and restorative planning.

## Figures and Tables

**Table 1:** Cephalometric analysis measurement values before pre-surgical orthodontic treatment.

Measurement		Normal Value	Value	Difference
Dental Analysis				
Interincisal angle	°	135.0	127.1	−7.9
+1/SN	°	102.0 ± 2.0	111.9	9.9
−1/MeGo (anatomic)	°	90.0 ± 3.0	89.1	−0.9
Skeletal Analysis				
N-S-Ar	°	123.0 ± 5.0	122.2	−0.8
Gonial angle (anatomic)	°	130.0 ± 7.0	122.4	−7.6
Bjork (anatomic)	°	396.0 ± 5.0	391.8	−4.2
Angle SNA	°	82.0 ± 2.0	79.7	−2.3
Angle SNB	°	80.0 ± 2.0	78.3	−1.7
ANB	°	2.0 ± 2.0	1.4	−0.6
Y/SN	°	66.0 ± 3.0	67.6	1.6
S-Go/N-Me	%	63.5 ± 1.5	66.6	3.1

**Table 2:** Cephalometric analysis measurement values at the end of the orthodontic treatment.

Measurement		Normal Value	Value Before Treatment	Value After Treatment	Difference
Dental Analysis					
Interincisal angle	°	135.0	127.1	130.5	3.4
+1/SN	°	102.0 ± 2.0	111.9	104.5	−7.4
−1/MeGo (anatomic)	°	90.0 ± 3.0	89.1	94.3	5.2
Skeletal Analysis					0
N-S-Ar	°	123.0 ± 5.0	122.2	121.2	−1
Gonial angle (anatomic)	°	130.0 ± 7.0	122.4	117.8	−4.6
Bjork (anatomic)	°	396.0 ± 5.0	391.8	390.7	−1.1
Angle SNA	°	82.0 ± 2.0	79.7	81.9	2.2
Angle SNB	°	80.0 ± 2.0	78.3	79.7	1.4
ANB	°	2.0 ± 2.0	1.4	2.2	0.8
Y/SN	°	66.0 ± 3.0	67.6	67.5	−0.1
S-Go/N-Me	%	63.5 ± 1.5	66.6	68.5	1.9

**Table 3:** Crown-to-root ratio of transplanted tooth in the 2.5 years post-surgery.

	Day of Surgery	1 m	3 m	6 m	9 m	1 y	1 y 3 m	1 y 9 m	2 y	2 y 6 m
**C:R** **Ratio**	1:1.03	1:0.99	1:1.16	1:1.23	1:1.40	1:1.52	1:1.49	1:1.83	1:1.81	1:1.94

**Table 4:** Cephalometric measurements taken before, soon after, and five years after autotransplantation.

Measurement		Normal Value	Before Treatment	After Treatment	5 Year Follow-Up
Dental Analysis					
Interincisal angle	°	135.0	127.1	130.5	132.4
+1/SN	°	102.0 ± 2.0	111.9	104.5	109.9
−1/MeGo (anatomic)	°	90.0 ± 3.0	89.1	94.3	95.5
Skeletal Analysis					
N-S-Ar	°	123.0 ± 5.0	122.2	121.2	115.9
Gonial angle (anatomic)	°	130.0 ± 7.0	122.4	117.8	117.9
Bjork (anatomic)	°	396.0 ± 5.0	391.8	390.7	382.2
Angle SNA	°	82.0 ± 2.0	79.7	81.9	89.5
Angle SNB	°	80.0 ± 2.0	78.3	79.7	86.6
ANB	°	2.0 ± 2.0	1.4	2.2	2.9
Y/SN	°	66.0 ± 3.0	67.6	67.5	59.7
S-Go/N-Me	%	63.5 ± 1.5	66.6	68.5	74.9

## Data Availability

The original contributions presented in this study are included in the article. Further inquiries can be directed to the corresponding author.

## Abbreviations

The following abbreviations are used in this manuscript:

ANB	A point–Nasion–B point (angle)	N	Nasion
Ar	Articulare	S	Sella
CT	Computed Tomography	SN	Sella–Nasion (plane)
Go	Gonion	SNA	Sella–Nasion–A point (angle)
Me	Menton	SNB	Sella–Nasion–B point (angle)
MeGo	Menton–Gonion line	Y	*Y*-axis (Sella to Gnathion)
ML	Mandibular Line		
